# Roles of AIM2 Gene and AIM2 Inflammasome in the Pathogenesis and Treatment of Psoriasis

**DOI:** 10.3389/fgene.2022.929162

**Published:** 2022-09-02

**Authors:** Jieyi Wang, Jing Gao, Cong Huang, Sohyun Jeong, Randy Ko, Xue Shen, Chaofeng Chen, Weilong Zhong, Yanfen Zou, Bo Yu, Changbing Shen

**Affiliations:** ^1^ Department of Dermatology, Peking University Shenzhen Hospital, Shenzhen, Guangdong, China; ^2^ Shenzhen Key Laboratory for Translational Medicine of Dermatology, Shenzhen Peking University—The Hong Kong University of Science and Technology Medical Center, Shenzhen, Guangdong, China; ^3^ School of Clinical Medicine, Health Science Center, Shenzhen University, Shenzhen, Guangdong, China; ^4^ Department of Dermatology, The Second Affiliated Hospital, Anhui Medical University, Hefei, Anhui, China; ^5^ Anhui Provincial Institute of Translational Medicine, Hefei, Anhui, China; ^6^ Marcus Institute for Aging Research at Hebrew SeniorLife, Boston, MA, United States; ^7^ Department of Medicine, Beth Israel Deaconess Medical Center and Harvard Medical School, Boston, MA, United States; ^8^ Department of Internal Medicine, University of New Mexico Health Sciences Center, Albuquerque, NM, United States; ^9^ Department of Dermatology, Chengdu Second People’s Hospital, Chengdu, Sichuan, China

**Keywords:** psoriasis, AIM2, AIM2 inflammasome, pathogenesis, treatment

## Abstract

Psoriasis is an immune-mediated chronic inflammatory skin disease caused by a combination of environmental incentives, polygenic genetic control, and immune regulation. The inflammation-related gene absent in melanoma 2 (*AIM2*) was identified as a susceptibility gene for psoriasis. AIM2 inflammasome formed from the combination of AIM2, PYD-linked apoptosis-associated speck-like protein (ASC) and Caspase-1 promotes the maturation and release of inflammatory cytokines such as IL-1β and IL-18, and triggers an inflammatory response. Studies showed the genetic and epigenetic associations between *AIM2* gene and psoriasis. *AIM2* gene has an essential role in the occurrence and development of psoriasis, and the inhibitors of AIM2 inflammasome will be new therapeutic targets for psoriasis. In this review, we summarized the roles of the *AIM2* gene and AIM2 inflammasome in pathogenesis and treatment of psoriasis, hopefully providing a better understanding and new insight into the roles of *AIM2* gene and AIM2 inflammasome in psoriasis.

## 1 Introduction

Psoriasis is an inflammatory skin disease caused by a combination of environmental incentives, polygenic genetic control, and immune regulation (Branisteanu et al., 2022). The worldwide average prevalence of psoriasis is 2% (Samotij et al., 2020), and the disease is often characterized by recurrence and incurability. It is often accompanied by complex comorbidities such as cardiovascular and autoimmune diseases (Gao et al., 2021), bringing great physical and mental harm to patients, and causing a substantial social–economic burden. The pathogenesis and novel therapies for psoriasis are hot research topics. In the past two decades, many susceptibility genes/loci and epigenetic modification factors of psoriasis have been identified (Nedoszytko et al., 2020). The inflammation-related gene absent in melanoma 2 (*AIM2*) was identified as a susceptibility gene for psoriasis (Zuo et al., 2015), and the function of *AIM2* gene and its role in psoriasis were explored (Ciążyńska et al., 2021; Liang et al., 2021). In this review, we systemically summarized genetic and epigenetic associations between *AIM2* gene and psoriasis, discussed the roles of *AIM2* gene and AIM2 inflammasome in the pathogenesis of psoriasis, and provided a better understanding of AIM2 inflammasome as a promising therapeutic target for psoriasis.

## 2 *AIM2* Gene and AIM2 Inflammasome

### 2.1 *AIM2* Gene

DeYoung et al. (1997) reported a novel gene, *AIM2*, also known as *PYHIN4*, located on chromosome 1q23.1-q23.2. The protein AIM2, encoded by the *AIM2* gene is a member of the IFI20X/IFI16 family, consisting of a C-terminal HIN domain and an N-terminal pyrin domain (PYD). RNA sequencing (RNA-seq) of 27 different human tissues showed that the expression level of *AIM2* gene in descending order are: lymph nodes, appendix, and spleen. *AIM2* gene is also expressed at a relatively low level in the skin compared with the top three tissues (Fagerberg et al., 2014). The expression of *AIM2* gene can be promoted by interferon-gamma (IFN-γ) (Tang H. et al., 2021). *AIM2* gene with various functions in the occurrence and development of diseases, the most common function is to initiate the assembly process of the AIM2 inflammasome. AIM2 response to dsDNA and then induce AIM2-dependent release of IL-18 and IL-1β, which plays a critical role as a trigger of autoimmune diseases, including psoriasis (Dombrowski et al., 2011), systemic lupus erythematosus (Shin et al., 2019), primary Sjogren’s syndrome (Vakrakou et al., 2020). *AIM2* gene plays two-sided roles in tumorigenesis or anti-tumorigenesis in different tumors (Choubey et al., 2000). *AIM2* gene with the tumor-promoting effects in nonsmall-cell lung cancer (NSCLC) via the inflammasome-dependent manner and regulation of mitochondrial dynamics (Qi et al., 2020). On the contrary, *AIM2* gene is required to restrain the progression of colon cancer through an inflammasome-independent manner, proliferation control of intestinal stem cells, and the regulation of gut microbiota, suggesting that *AIM2* gene plays a protective role in colorectal cancer (Wilson et al., 2015; Zhang Z. et al., 2017).

### 2.2 AIM2 Inflammasome

In 2009, the composition of the AIM2 inflammasome was reported (Fernandes-Alnemri et al., 2009; Hornung et al., 2009). AIM2 can recognize double-stranded DNA (dsDNA) and interacts with N-terminal PYD. PYD-linked apoptosis-associated speck-like protein (ASC) induces the recruitment of Caspase-1 by the caspase recruitment domain (CARD) of ASC to form the AIM2 inflammasome. AIM2 inflammasome can induce the maturation and release of inflammatory factors such as IL-1β and IL-18, triggering an inflammatory response (Wang et al., 2019). Studies over the past decades have shown that AIM2 inflammasome plays a vital role in many kinds of inflammatory diseases, immune diseases, and cancers (Man et al., 2016; Kumari et al., 2020; Zhu H. et al., 2021).

**FIGURE 1:**
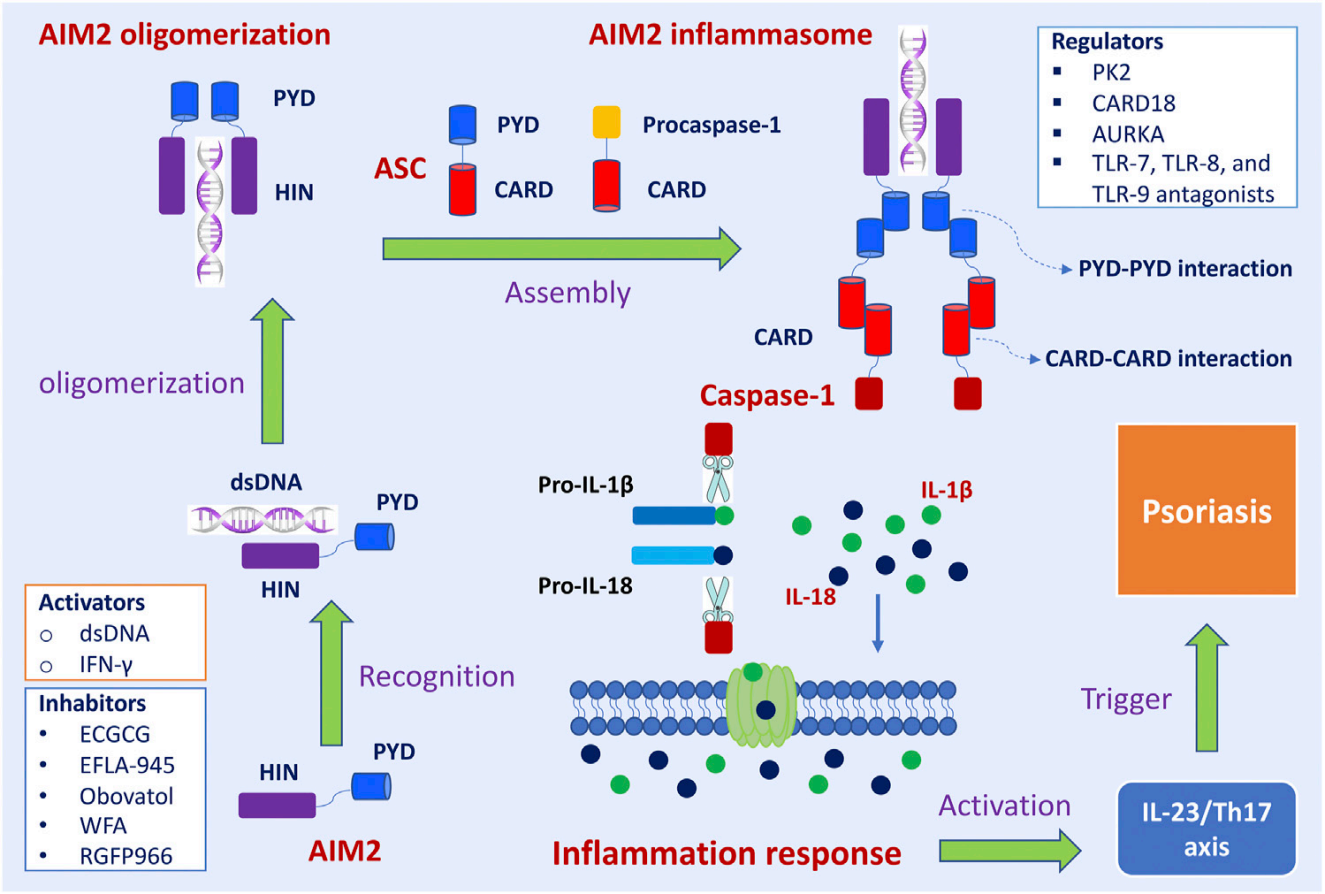
The assembly and activation of AIM2 inflammasome and subsequently regulatory and trigger pathways in psoriasis. Upon sensing viral DNA, self-derived dsDNA, and cytosolic bacterium, the HIN domain of AIM2 directly recognizes dsDNA in a sequence-independent manner, which triggers the assembly of the AIM2 oligomerization. The PYD domain of AIM2 interacts with the PYD of a recruiting adapter protein ASC, resulting in a high polymer complex AIM2 inflammasome. Inactive procaspases-1 are recruited into AIM2 inflammasome via the CARD-CARD interaction. When the main components of the inflammasome are connecting and the active inflammasome is formed, it directly recruits and cleaves pro-caspase1 into active caspase-1, which proteolytically activates the pro-inflammatory cytokines IL-1β and IL-18. These inflammatory cytokines directly induce inflammatory responses and participate in the occurrence and development of diseases. The active IL-1β and IL-18 involve in IL23/Th17 pathway and then induce many kinds of chemokines and inflammatory cytokines, which trigger the development of psoriasis. Interferon-gamma (IFN-γ) promoted the expression of the *AIM2* gene. Epigallocatechin gallate (EGCG), EFLA-945, obovatol, withaferin A (WFA), and RGFP966 are inhibitory effects on AIM2. Prokineticin 2 (PK2), caspase recruitment domain family member 18 (CARD18), aurora kinase A (AURKA), TLR-7, TLR-8, and TLR-9 antagonists are regulators of AIM2 inflammasome signaling pathway.

The assembly and activation of AIM2 inflammasome and subsequent inflammatory response was shown in Figure 1. The primary role of AIM2 is to initiate the assembly process of the inflammasome. ASC acts as an inflammasome adaptor protein, connecting upstream AIM2 and downstream Caspase-1. Caspase-1 is the effector protein of AIM2 inflammasome, and the activated caspase-1 leads to proteolytic cleavage of IL-1β and IL-18. The massive release of downstream inflammatory cytokines (IL-1β and IL-18) directly affects the host’s innate immune regulation to infection and injury, induces acute and chronic inflammatory responses, and participates in the occurrence and development of these diseases, such as skin diseases, chronic kidney disease, cardiovascular diseases, neuronal diseases, and diabetes mellitus (Sharma et al., 2019).

## 3 Genetic and Epigenetic Associations Between *AIM2* Gene and Psoriasis

### 3.1 Genetic Associations Between *AIM2* Gene and Psoriasis

In the early stage, sequencing results showed that the coding region of *AIM2* gene with a high frequency of frameshift and missense mutations in primary high-level microsatellite instability (MSI-H) colon cancers and cell lines (Woerner et al., 2007). In 2015, an exome-wide association study in large-scale individuals was performed to investigate the coding variants in psoriasis. *AIM2* gene was firstly identified as a susceptibility gene for psoriasis at the genome-wide level, this gene locates at an important topological position in the gene–gene interaction network. In addition, Zuo et al. (2015) predicted that a variant (rs2276405) in *AIM2* gene affects AIM2 protein structure (Glu32Lys). The chemical properties of Glu (the chemical nature of Glu residue is acidic) and Lys (the chemical nature of Lys residue is alkaline) are completely opposite, which may destabilize the alpha-helix motif. Researchers investigated the correlation between the genetic pattern of *AIM2* gene polymorphism and the psoriasis phenotype (Li et al., 2016). Genotype and allele distribution of *AIM2* gene showed that allele A was the minor allele and G was the risk allele. Genetic pattern analysis showed that the dominant pattern was the best genetic pattern for *AIM2* gene polymorphism loci. Compared with the controls, the distribution of the dominant inheritance pattern was higher in psoriasis area and severity index (PASI) score ≤20 than that of PASI score >20, and family history of negative patients is more significantly different statistically than positive family history.

### 3.2 Epigenetic Associations Between *AIM2* Gene and Psoriasis

Epigenetic associations between *AIM2* gene and diseases have also been studied. Hypermethylation of the *AIM2* promoter conferred insensitivity to IFN-γ-induced *AIM2* expression of MSI-H colon cancer cell lines, demonstrating the inactivation of *AIM2* was regulated by epigenetic factors (Woerner et al., 2007). In 2016, an epigenome association study (EWAS) of psoriasis was conducted in the Chinese Han population, and the results showed that three CpG sites (cg17217296, cg17515347, and cg07195224) in the promoter region of *AIM2* gene were significantly associated with psoriasis (Li et al., 2016). Assay for transposase-accessible chromatin using sequencing (ATAC-seq) was used to explore the landscape of chromatin accessibility of psoriatic skin tissue (PP) and nonpsoriatic skin tissue (PN) from patients with psoriasis and normal skin tissue (NN) from healthy individuals, the results show that *AIM2* gene promoter region was specifically more accessible in PP, and contained a CpG site (cg07195224), which was previously reported to be significantly hypomethylated in psoriasis (Tang L. et al., 2021). In addition, it was observed that the intensity of the promoter-associated peak of *AIM2* was negatively correlated with the methylation level of cg07195224 but positively correlated with *AIM2* mRNA expression level. Meanwhile, the methylation level of cg07195224 was strongly and negatively associated with *AIM2* mRNA expression level (Tang L. et al., 2021). Studies will be needed to explore the correlation between epigenetic regulation and *AIM2* gene expression in the future and to further clarify its regulating effect in the pathogenesis of psoriasis.

## 4 Roles of *AIM2* Gene and AIM2 Inflammasome in the Pathogenesis of Psoriasis

### 4.1 AIM2 Inflammasome Mediated Inflammatory Response Involved in Psoriasis

The roles of *AIM2* gene in the pathogenesis of psoriasis have been studied (Table 1). AIM2 inflammasome activity may represent a potential trigger for the occurrence and development of inflammatory diseases. Kopfnagel et al. (2011) found that the AIM2 inflammasome is active in human keratinocytes, triggering IL-1β secretion with an important role in inflammatory processes. In cultured keratinocytes, the expression of *AIM2* gene was induced by INF-γ; cytoplasmic DNA can trigger the release of IL-1β through the AIM2 inflammasome (Dombrowski et al., 2011). In CD14^+^ and CD16^+^ monocyte subsets in the blood of patients with psoriasis, the *AIM2* gene expression level was significantly higher than that of the control group, indicating that AIM2 inflammasome exists in the immune cell subsets in the peripheral blood of psoriasis patients in a stimulated state (Verma et al., 2021).

**TABLE 1:** Roles of *AIM2* gene and AIM2 inflammasome in the pathogenesis of psoriasis.

Studies/Year	Objects	Main findings
Kopfnagel et al. (2011)	Keratinocyte	AIM2 inflammasome is active in human keratinocytes and triggers IL-1β secretion, which represents a potential trigger factor for the development and maintenance of inflammatory skin diseases
Dombrowski et al. (2011)	Skin tissue, Keratinocyte	Abundant cytoplasmic DNA and increased *AIM2* expression were detected in psoriatic skin lesions; *AIM2* expression was induced by INF-γ in cultured keratinocytes, and cytoplasmic DNA can trigger the release of IL-1β through the AIM2 inflammasome
de Koning et al. (2012)	Skin tissue	AIM2 protein expression is significantly upregulated in the psoriatic epidermis. *AIM2* expression was dynamics in human tissues and primary cells, restricted expression in Langerhans cell and melanocyte of the normal epidermis, but with a strong upregulation in subpopulations of epidermal keratinocytes under inflammatory conditions
Göblös et al. (2016)	Keratinocyte	Gene-specific silencing of *CARD18* in cells treated with poly (dA:dT) resulted in a significant decrease in *AIM2* gene expression and significantly reduced *Caspase-1* mRNA expression, which indicates that *CARD18* might indeed contribute to the fine-tuning of keratinocyte innate immune processes
Šahmatova et al. (2017)	Skin tissue	*AIM2* gene was expressed at an increased level in psoriatic skin
Yuan et al. (2020)	Skin tissue	High expression of *AIM2* gene can be detected in the epidermis of psoriatic skin lesions. Neutrophil extracellular trapping net (NET) may promote the expression of *AIM2* gene by activating keratinocytes. The secretion of IL-1β accelerates the inflammatory process of psoriasis
Verma et al. (2021)	Blood	An increased *AIM2* expression in the CD14^+^ and CD16^+^ subsets of patients was observed, suggesting that *AIM2* exists in a primed state in the immune cell subsets in the peripheral blood of the psoriasis patients
Tang H. et al. (2021)	Skin tissue, Keratinocyte	AURKA promotes the occurrence and development of psoriatic inflammation by blocking autophagy-mediated suppression of the AIM2 inflammasome
Zhao et al. (2022)	Skin tissue	The AIM2 total fluorescence intensity in CD4^+^ Trm cells in patients with SCLE and localized DLE was higher than in patients with psoriasis. The expression of *AIM2* gene in skin CD4^+^ Trm cells can be a significant indicator to distinguish patients with ACLE from those with localized DLE and SCLE.

The expression of *AIM2* gene in lesional skin from psoriasis patients was explored in several studies (Dombrowski et al., 2011; de Koning et al., 2012; Šahmatova et al., 2017; Yuan et al., 2020). Compared with skin tissues from healthy individuals, abundant cytoplasmic DNA and increased *AIM2* gene expression were detected in psoriatic skin lesions (Dombrowski et al., 2011). Šahmatova et al. (2017) also identified that the *AIM2* gene was expressed at an increased level in psoriatic skin lesions. Yuan et al. (2020) found that the formation of neutrophil extracellular trapping net (NET) structure and the high expression of *AIM2* gene can be detected in the epidermis of skin lesions from psoriasis patients. NET may promote the expression of *AIM2* gene by activating keratinocytes, and the secretion of IL-1β accelerates the inflammatory process of psoriasis. In addition, the expression of *AIM2* gene is also significantly upregulated in several inflammatory skin disorders, including contact dermatitis, venous ulcers, atopic dermatitis, and experimental wounds (de Koning et al., 2012). The expression of *AIM2* gene was dynamic in human tissues and primary cells, with restricted expression in Langerhans cell and melanocytes of the normal epidermis, but a strong upregulation in subpopulations of epidermal keratinocytes under inflammatory conditions (de Koning et al., 2012).

The IL-23/Th17 axis plays a central role in the development of psoriasis (Hawkes et al., 2017), some biologics (IL-17 antagonists: Secukinumab, Ixekizumab, and Brodalumab; IL-23 antagonists: Risankizumab, Guselkumab, and Tildrakizumab) focus on this signaling pathway have been used for the treatment of moderate-severe psoriasis (Sharma et al., 2022). AIM2 induces AIM2-dependent release of IL-18 and IL-1β; furthermore, the active IL-1β and IL-18 involve in IL23/Th17 pathway and then induce many kinds of chemokines and inflammatory cytokines (Ciążyńska et al., 2021). Taken together, the aforementioned studies indicate that the activation of the AIM2 inflammasome may be a potential trigger for the development of psoriasis, and the inflammatory response mediated by the AIM2 inflammasome plays an important role in the initial onset and persistence of psoriasis.

### 4.2 Factors Regulate AIM2 Inflammasome Signaling Pathway in Psoriasis

Recent studies have found that certain factors regulate the AIM2 inflammasome signaling pathway and participate in the pathogenesis of psoriasis, including prokineticin 2 (PK2), caspase recruitment domain family member 18 (CARD18), aurora kinase A (AURKA), TLR-7, TLR-8, and TLR-9 antagonists.

#### 4.2.1 PK2

PK2 is a psoriasis-specific factor that is highly expressed in mouse and human psoriatic skins, but is not significantly expressed in other autoimmune diseases, such as inflammatory bowel diseases, atherosclerosis, and diabetes; PK2 significantly increases the expression of Caspase-1, and also strongly up-regulates the AIM2 inflammasome signaling pathway in monocytic THP-1 cells, which is engaged by AIM2 to promote the synthesis and secretion of proinflammatory cytokine IL-1β (He et al., 2016). Thus, PK2 regulates the AIM2 inflammasome signaling pathway involved in the pathogenesis of psoriasis.

#### 4.2.2 CARD18

CARD18 is highly expressed in psoriatic noninvolved epidermis compared to healthy skin epidermis (Szabó et al., 2014). CARD18 involves AIM2 inflammasome-mediated keratinocyte functions and modifies the inflammatory process in keratinocytes. Silencing of *CARD18* in keratinocyte cells treated with poly (dA:dT) resulted in a significant decrease in *AIM2* gene expression and significantly reduced *Caspase-1* mRNA expression (Göblös et al., 2016), which indicates that CARD18 may contribute to the fine-tuning of innate immune processes.

#### 4.2.3 AURKA

AURKA is a member of the serine/threonine kinases family, which plays a critical role in the suppression of autophagy (Zhang S. et al., 2017), and is elevated in lesional psoriatic tissue (Liu et al., 2011). A recent study found that AURKA promotes the occurrence and development of psoriatic inflammation by blocking autophagy-mediated suppression of the AIM2 inflammasome (Tang H. et al., 2021).

#### 4.2.4 TLR-7, TLR-8, and TLR-9 Antagonists

Immune modulatory oligonucleotides and small molecular weight compounds, IMO-3100, IMO-8400, and IMO-9200 target TLR-7, TLR-8, and TLR-9, respectively. These are under clinical investigation for their effectiveness in the treatment of psoriasis. Chemical compounds, such as AS-2444697, PF-05387252, PF-05388169, PF-06650833, ML120B, and PHA-408, can inhibit TLR signaling (Gao et al., 2017). Jiang et al. (2013) evaluated an antagonist of TLR-7, TLR-8, and TLR-9 as a therapeutic agent in an IL-23-induced psoriasis model in C57BL/6 mice. Treatment with an antagonist reduced the expression of inflammasome components (including NLRP3, AIM2, and antimicrobial peptides) in the dermis, which indicated that targeting TLR-7, TLR-8, and TLR-9 may provide a method for neutralizing the multiple inflammatory pathways that are involved in psoriasis.

### 4.3 How Fra-1 Regulates AIM2 and What’s the Regulatory Effect in Psoriasis?

Recently, bioinformatics prediction and luciferase reporter assay showed that Fra-1 targeted binding to 363 bp and 57 bp upstream of the *AIM2* gene transcription start site (Tang L et al., 2021). Fra-1 is encoded by the *FOSL1* gene and plays a role in cell proliferation and differentiation, gene expression and regulation, and the occurrence and progression of psoriasis (Talotta et al., 2020). *FOSL1* knockdown inhibited IL-22-induced proliferation and enhanced keratinocyte apoptosis, whereas IL-22 stimulation and *FOSL1* overexpression further enhanced keratinocyte proliferation (Meng et al., 2021). The expression of *FOSL1* was significantly increased in lesional psoriatic skin and positively correlated with the PASI score (Sobolev et al., 2011). The aforementioned findings show that the high expression of *FOSL1* in lesional psoriatic skin is one of the markers of the pathological activity of psoriasis and that Fra-1 plays an important role in the pathogenesis of psoriasis. However, Fra-1 needs to be further investigated on how it regulates the expression of *AIM2* in psoriasis, which is still unclear.

### 4.4 The Expression of *AIM2* Gene Can Be a Biomarker to Distinguish Different Subtypes of Psoriasis?

The expression level of *AIM2* gene in CD4^+^ tissue-resident memory T (CD4^+^ Trm) cells was measured in the patients with acute cutaneous lupus erythematosus (ACLE), subacute CLE (SCLE), localized discoid lupus erythematosus (localized DLE), psoriasis, and other inflammatory skin diseases (Zhao et al., 2022). The results showed that *AIM2* expression in CD4^+^ Trm cells was significantly lower in patients with ACLE than in localized DLE and SCLE. In psoriasis patients, CD4^+^ Trm cells were mainly located in the epidermis of skin lesions. The AIM2 total fluorescence intensity in CD4^+^ Trm cells in patients with SCLE and localized DLE were higher than in patients with psoriasis. Compared to ACLE with localized DLE and/or SCLE, the receiver operating characteristic curve for *AIM2* expression in CD4^+^ Trm cells had different sensitivities and specificities at different cutoff values. Therefore, *AIM2* expression in skin CD4^+^ Trm cells can be a significant indicator of distinguishing patients with ACLE from those patients with SCLE and localized DLE. Psoriasis is also divided into subtypes with different clinical manifestations, including psoriasis vulgaris, erythrodermic psoriasis, pustular psoriasis, and psoriatic arthritis (Zhu C. et al., 2021). It is worthy to further investigate whether *AIM2* expression can be a biomarker to distinguish different subtypes of psoriasis. A valuable study will be carried out in the future to explore this scientific question.

## 5 AIM2 Inflammasome Is a Promising Therapeutic Target for Psoriasis

### 5.1 AIM2 Correlates With Therapeutic Efficacy of Psoriasis

Recent studies have shown that the expression of *AIM2* gene can be a biomarker to predict the benefit of therapy in patients with epithelial ovarian cancer (Hsu et al., 2021), melanoma (Fukuda et al., 2021), systemic lupus erythematosus (Yang et al., 2021) and heart failure (Onódi et al., 2021). These studies implicate its potential utility in predicting clinical treatment outcomes. Gong et al. calculated innate immune cell proportion in psoriatic skin by utilizing microarray data, results show that *AIM2* gene was negatively associated with resting mast cells but positively associated with activated dendritic cells (Gong and Wang, 2021). Brodalumab and Ustekinumab are used to treat moderate to severe plaque psoriasis, and most patients can get better treatment effects. Interestingly, *AIM2* gene was positively associated with the therapeutic efficacy of Brodalumab and negatively associated with Ustekinumab treatment response (Gong and Wang, 2021), which provide novel clues for clinical decisions on treatment for psoriasis. In addition, KEGG analysis shows pathways involving RIG-I-like receptor signaling, NOD-like receptor signaling, toll-like receptor signaling, and cytosolic DNA-sensing pathways, these pathways were significantly enriched and positively correlated with *AIM2* gene (Gong and Wang, 2021).

### 5.2 LL-37 Neutralizes Inflammation Mediated by AIM2 Inflammasome

The antimicrobial peptide LL-37 (also known as cathelicidin antimicrobial peptide, CAMP) is overexpressed in psoriatic lesions and acts as the critical factor that mediates plasmacytoid dendritic cells (pDCs) activation in psoriasis (Lande et al., 2007). AIM2 inflammasomes activate pro-inflammatory processes and trigger IL-1β secretion in psoriatic lesions. However, IL-1β secretion was completely abolished when LL-37 and DNA were delivered together into keratinocytes. This may suggest that LL-37 can translocate into the cytosol of psoriatic keratinocytes and specifically neutralizes cytosolic DNA, thus acting as an inhibitor of AIM2 inflammasome activation (Dombrowski and Schauber, 2012). Topical treatment with vitamin D analogs decreases inflammation and pro-inflammatory cytokines, but strongly increases cathelicidin’s expression (Lebwohl et al., 2004; Peric et al., 2009). However, repeated treatments with narrowband-UVB (NB-UVB) decreased skin inflammation in patients with psoriasis but increased vitamin D serum levels and the expression of cutaneous cathelicidin (Vähävihu et al., 2010). Thus, established therapies targeting the vitamin D pathway reduce inflammatory responses while increasing epidermal antimicrobial peptide expression in psoriatic lesions, the anti-inflammatory effect of LL-37 on the AIM2 inflammasome pathway may account for these observed effects (Reinholz et al., 2012). LL-37 can modulate the pathogenic response to nucleic acids and be helpful for the development of anti-inflammatory therapies.

### 5.3 Potential AIM2 Inflammasome Inhibitors for Psoriasis Treatment

At present, there are some AIM2 inflammasome inhibitors that have been explored to treat inflammatory diseases, including epigallocatechin gallate (EGCG), EFLA-945, obovatol, withaferin A (WFA), and RGFP966.

#### 5.3.1 EGCG

EGCG is the most abundant main polyphenol component of green tea, and the alloy moiety of catechins possesses the most biological activities, including angiogenesis and anti-inflammatory effects (Kondo et al., 2002). The structure of EGCG and related information are shown in Supplementary Figure S1. EGCG inhibits the transfection of NF-κB and AP-1 to downregulate the expression of reactive oxygen species (ROS) and reactive nitrogen species (RNS) and decreases the production of inflammatory factors (Nagai et al., 2002). Studies have shown that EGCG attenuates AIM2-induced IL-1β secretion by inhibiting inflammasome IFN-γ secretion and dA:dT-induced ASC oligomerization in neonatal human epidermal keratinocytes (Yun et al., 2015).

#### 5.3.2 EFLA-945

EFLA-945 is an extract from red grapevine leaf in traditional medicine in Japan (Rabe et al., 2011) and over-the-counter drugs in Europe and Japan (Hoshino et al., 2018) for its anti-inflammatory properties and circulatory benefits. Resveratrol (Supplementary Figure S2) and peonidin 3-O-glucoside (Supplementary Figure S3) are the major phytochemicals of EFLA 945. Study results showed that EFLA-945 could limit the entry of DNA into THP-1-derived macrophages, thereby inhibiting cytoplasmic DNA-dependent ASC and activation of the AIM2 inflammasome. Resveratrol and peonidin 3-O-glucoside are two major phytochemicals of EFL-945 that mediate this inhibition (Chung et al., 2020). Furthermore, EFLA-945 attenuated the associated pro-inflammatory response in localized skin lesions of an imiquimod-induced psoriasis-like mouse model, indicating that EFLA-945 may be beneficial for the treatment of psoriasis.

#### 5.3.3 Obovatol

Obovatol (Supplementary Figure S4), a bisphenol chemical originating from Magnolia obovata, affects the AIM2 inflammasome by inhibiting the formation of ASC pyroptosome and the generation of mitochondrial ROS. Furthermore, obovatol has been used as a traditional treatment for inflammatory diseases (Kim et al., 2019) and neuroinflammation (Ock et al., 2010). Furthermore, obovatol disrupted inflammasome activation’s initiation step, inhibited the transcription of inflammatory cytokines, and decreased serum IL-1β elevation in response to sodium urate crystals in mice (Kim et al., 2019). Whether obovatol has a therapeutic effect on psoriasis will be further studied.

#### 5.3.4 WFA

WFA is an extract from the medicinal plant *Withania somnifera* and has various biological activities, including acting as an anti-inflammatory, angiogenesis, and anticancer (Mohan et al., 2004). The structure of EGCG and related information are shown in Supplementary Figure S5. Studies show that WFA regulates AIM2 inflammasome and Caspase-1 in THP-1 polarized macrophages; however, WFA treatment of M2 macrophages inhibits TGF-β compared with M1 secretion (Ngoungoure and Owona, 2019). Currently, further research is needed to determine whether WFA can affect the occurrence and progression of psoriasis by inhabiting the AIM2 inflammasome pathway.

#### 5.3.5 RGFP966

Histone deacetylases 3 (HDAC3) modulates the acetylation of histone and non-histone proteins. RGFP966 (Supplementary Figure S6) is a selective inhibitor of HDAC3 (Zhang et al., 2020). Specifically, RGFP966 regulates the inflammatory process in stroke (Chen et al., 2012) and brain damage (Zhang et al., 2020). Lipopolysaccharide (LPS) stimulation caused time-dependent increases of HDAC3 and AIM2 inflammasome in primary cultured microglia. *AIM2* gene was spatiotemporally regulated by RGFP966, which was confirmed in an experimental mouse stroke model. RGFP966 can enhance STAT1 acetylation and decrease STAT1 phosphorylation, which may partially explain the negative regulatory effect of *AIM2* gene by RGFP966 (Zhang et al., 2020). This study indicated that RGFP966 alleviated the inflammatory process by regulating the AIM2 inflammasome. The further therapeutic effects of RGFP966 on psoriasis by regulating the AIM2 inflammasome will be investigated in the future.

## 6 The Disadvantages of Targeting *AIM2* Gene as a Potential Therapeutic Target

*AIM2* gene act as a double-edged sword in the pathogenesis of some autoimmune diseases and cancers. The increased and decreased expression of *AIM2* gene with different roles in the development of different diseases. *AIM2* gene plays the tumor-suppressive role in HPV-infected cervical cancer (So et al., 2018), breast cancer (Yoon et al., 2015), squamous cell carcinoma (Farshchian et al., 2017); while *AIM2* gene is regarded as a protective factor in melanoma (DeYoung et al., 1997; de Koning et al., 2014) and colorectal cancer (Wilson et al., 2015; Zhang Z. et al., 2017). Intriguingly, *AIM2* gene with two contrasting roles in different models or different disease stages of hepatocellular carcinoma (Martínez-Cardona et al., 2018; Shi et al., 2019). *AIM2* gene as a therapeutic target may with different effects on different diseases, therefore we should consider the advantages and disadvantages of targeting *AIM2* gene. For instance, when we use AIM2 inflammasome inhibitors or drugs to treat psoriatic patients also with cancer, in the future, we need to pay attention to its impact on cancer. Taken together, the advantages and disadvantages of targeting *AIM2* gene should be considered at the same time.

## 7 Conclusion and Perspectives

In this review, we provided an overview of the research progress on the effect and role of *AIM2* gene and AIM2 inflammasome in psoriasis. We hope this provides further potential therapeutic targets for psoriasis. Although some potential inhibitors of the AIM2 inflammasome were explored, no AIM2 inflammasome target drug has been used in the clinical treatment of psoriasis. Therefore, further researches need to focus on the development of new drugs for psoriasis.

## Fundings

This study was supported by grants from the Research Foundation of Peking University Shenzhen Hospital (No: JCYJ2020004), Shenzhen Sanming Project (No: SZSM201812059), Shenzhen Key Medical Discipline Construction Fund (No: SZXK040), Guangdong Basic and Applied Basic Research Foundation (No: 2021A1515110097), National Natural Science Foundation of China (No: 82103726), Natural Science Foundation of Anhui Province (No: 2008085QH427), and Research Fund of Anhui Institute of Translational Medicine (No: 2021ZHYX-C48).
