# Supplementary material for: Roles of AIM2 Gene and AIM2 Inflammasome in the Pathogenesis and Treatment of Psoriasis
Source: Front Genet. 2022 Sep 2;13:929162. doi: 10.3389/fgene.2022.929162 (PMC9481235; doi:10.3389/fgene.2022.929162)
Supplement: Supplementary file 1 [file DataSheet1.pdf]

Supplementary materials

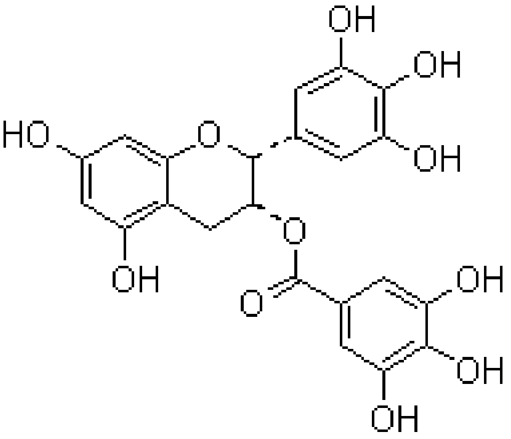

Supplementary Figure S1 | Structure of epigallocatechin gallate

(Synonyms: (2R,3R)-2-(3,4,5-Trihydroxyphenyl)-3,4-dihydro-1(2H)-benzopyran-3,5,7-triol 3-(3,4,5-trihydroxybenzoate); EGCG. Molecular formula: C22H18O11. Molecular weight: 458.38 Da. CAS registry number: 989-51-5. Information and figure of epigallocatechin gallate are from https://www.chemblink.com/index.htm)

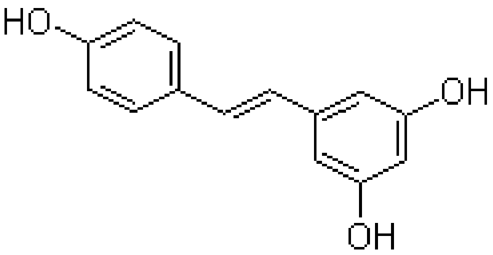

Supplementary Figure S2 | Structure of resveratrol

(Synonyms: 3,4',5-Trihydroxy-trans-stilbene; 5-[(1E)-2-(4-Hydroxyphenyl)ethenyl]-1,3-benzenediol. Molecular formula: C14H12O3. Molecular weight: 228.24. CAS registry number: 501-36-0. Information and figure of resveratrol are from https://www.chemblink.com/index.htm)

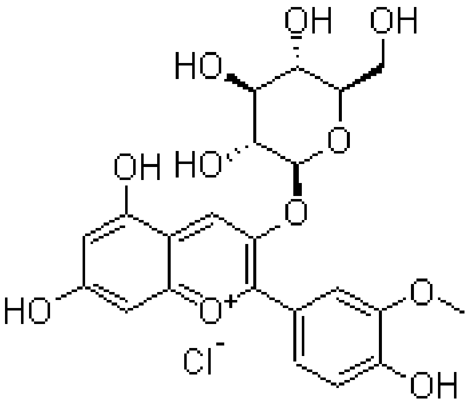

Supplementary Figure S3 | Structure of peonidin 3-O-glucoside

(Synonyms: 3-(beta-D-Glucopyranosyloxy)-5,7-dihydroxy-2-(4-hydroxy-3-methoxyphenyl)-1-benzopyrylium chloride. Molecular formula: C22H23O11.Cl. Molecular weight: 498.86.

CAS registry number: 6906-39-4. Information and figure of peonidin 3-O-glucoside are from https://www.chemblink.com/index.htm)

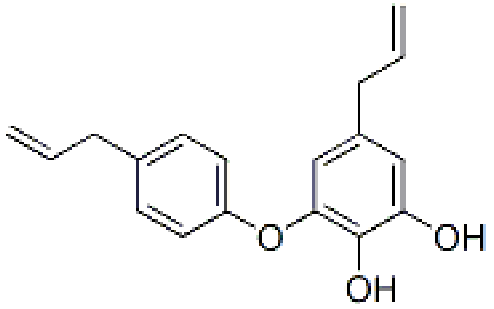

Supplementary Figure S4 | Structure of obovatol

(Synonyms: 5-Allyl-3-(4-Allylphenoxy)Benzene-1,2-Diol; 5-Allyl-3-(4-Allylphenoxy)Pyrocatechol; Obovatol. Molecular formula: C18H18O3. Molecular weight: 282.34 Da. CAS registry number: 83864-78-2. Information and figure of obovatol are from https://www.chemblink.com/index.htm)

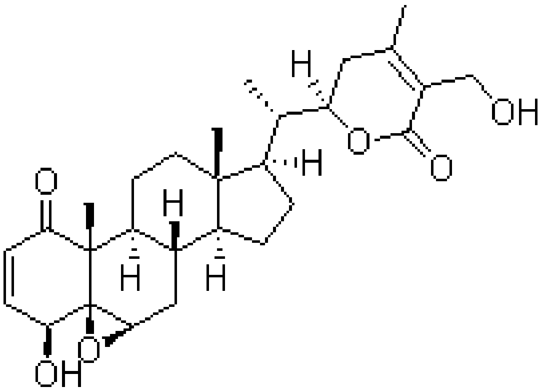

Supplementary Figure S5 | Structure of withaferin A

(Synonyms: Withaferine; NSC 101088; NSC 273757. Molecular formula: C28H38O6. Molecular weight: 470.60 Da. CAS registry number: 5119-48-2. Information and figure of withaferin A are from https://www.chemblink.com/index.htm)

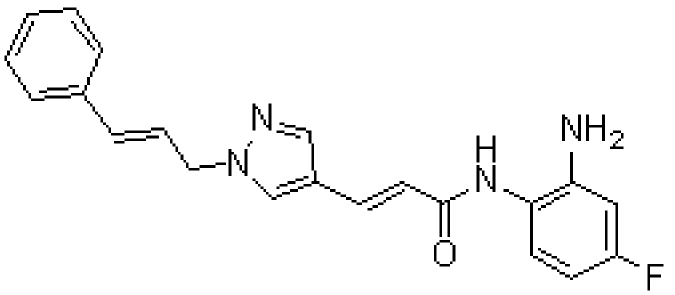

Supplementary Figure S6 | Structure of RGFP966

(Synonyms: (2E)-N-(2-Amino-4-fluorophenyl)-3-[1-(3-phenyl-2-propen-1-yl)-1H-pyrazol-4-yl]-2-propenamide. Molecular formula: C21H19FN4O. Molecular weight: 362.40 Da. CAS registry number: 1357389-11-7. Information and figure of RGFP966 are from https://www.chemblink.com/index.htm)
